# Analysis of TERT Rs2736100 genotype distribution in laryngeal squamous cell carcinoma patients

**DOI:** 10.25122/jml-2022-0114

**Published:** 2022-09

**Authors:** Corina Iulia Cornean, Alma Aurelia Maniu

**Affiliations:** 1 Department of Otolaryngology-Head and Neck Surgery, Iuliu Hatieganu University of Medicine and Pharmacy, Cluj-Napoca, Romania

**Keywords:** laryngeal squamous cell carcinoma, PCR analysis, TERT Rs2736100, ETDA – ethylenediaminetetraacetic-acid, G1 – well-differentiated squamous cell carcinoma, G2 – moderately differentiated squamous cell carcinoma, G3 – poorly differentiated squamous cell carcinoma, HNC – head and neck cancer, LSCC – laryngeal squamous cell carcinoma, M – distant metastasis, N – regional node metastasis, Real-time-PCR – Real-time polymerase chain reaction, SCC – squamous cell carcinoma, TERC – telomere ARN component, TERT – telomerase reverse transcriptase, TNM – tumor classification criteria-T (the primary tumor), UADT – upper aerodigestive tract

## Abstract

The applicability of the telomerase reverse transcriptase (TERT) gene Rs2736100 polymorphism in cancer research has been well documented for various malignancies except for head and neck cancers, where data is sparse. This study aimed to analyze this polymorphism with the pathological characterizers of laryngeal squamous cell carcinoma (LSCC) patients. Genetic testing was performed using the Real-time PCR technique on 56 paired samples of biological material (blood and tissue). Data were analyzed using Epi info 7 software. The subjects were predominantly male (95% *vs*. 5%), with a median age of 62 years, and smokers (89%). The primary tumor origin site was the glottic region (34%), and the advanced clinical stages III-IV were more common (46% *vs*. 18%). Results show high frequencies for the mutated variants of Rs2736100 (CC 36%>AC 34%>AA 30%), while distribution according to tumor classification criteria leaned towards moderately differentiated carcinoma specimens in T3-T4 stages for the AC/CC variants (P-value without statistical significance) but positively favored the relationship between the AA variant and lack of lymph node metastasis (P=0.0106). The genotypes tend to associate themselves with a better histological presentation regarding the pattern of tumor invasion and, thus, better prognostic values for LSCC. Results suggest that the wild-type genotype of TERT Rs2736100 may be a protective factor for lymph node metastasis and histological pattern of tumor invasion in LSCC. Results regarding the synergistic relationship between cancer and smoking corroborate literature data for moderate to severe smokers regardless of the genetic variant.

## INTRODUCTION

**Figure 1:**
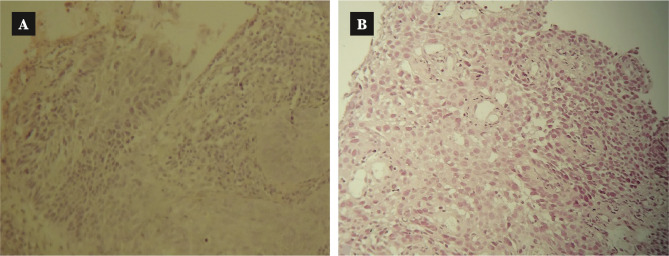
Histological grade differentiation of squamous cell carcinoma (SCC) in hematoxilin-eozinofil (HE) staining. A – Well differentiated SCC closely resembling normal squamous epithelium (basal-type cells and squamous cells with keratinization and intercellular bridges; pleomorphic hyperchromatic nuclei; very rare atypical mitoses). B – Poorly differentiated SCC (predominance of immature cells with minimal keratinisation and intercellular bridges, numerous atypical mitoses).

Although considered to be the second most common neoplasm amongst upper aerodigestive tract (UADT) malignancies, more than 85–95% of laryngeal cancer forms are represented by squamous cell carcinoma (SCC) derived from the epithelial lining (stratified squamous epithelium/respiratory epithelium) of the larynx. The transformation occurs due to squamous metaplasia secondary to variable influential risk factors. The defining hallmark of SCC attributed to squamous differentiation (Figure 1) is represented by the presence of keratin formation and/or the presence of intercellular bridges to various degrees [1, 2]. Although the incidence rate according to primary tumor location varies according to different populations and the anatomical sections of the larynx, the most notable are the supraglottic and glottic regions. Concurrently, the evolutionary behavior of this pathology simultaneously affects the dual function of the larynx in terms of both breathing and speaking, thus making it one of the most life-altering pathologies [2, 3].

The telomerase enzyme is a vital factor in maintaining the ends of telomeres located at the termini of eukaryotic chromosomes [4]. Telomeres are heterochromatic domains composed of tandem repeats of a DNA sequence rich in G bases, TTAGGG in all vertebrates, [5, 6] bound to an array of specialized proteins (a six-protein complex) known as shelterin [7] who protect chromosomes from unscheduled DNA repair and degradation [8]. During the cell cycle, the inability of DNA polymerase to completely replicate the 3' ends of chromosomal DNA during each S phase has been linked to the progressive shortening of telomeres [9]. The erosion, caused by successive replication cycles, eventually ensures the loss of the ability to protect the ends of chromosomal DNA. This process forces them to engage in end-to-end chromosomal fusions giving way to karyotypic disarray and resulting in the inevitable death of the cell [6]. Both the length of the telomere repeats as well as the integrity of the telomere-binding proteins are essential in ensuring telomere protection, as telomere dysfunction may be sufficient in causing premature tissue degeneration, acquisition of chromosomal aberrations, and initiation of neoplastic lesions [8].

In humans, two components of the telomerase enzyme ensure the reconstruction process for telomere activity. Both TERT (telomerase reverse transcriptase) and TERC (the RNA component) components work to synthesize tandem repeats of the tetrahymena telomeric sequence d(TTGGGG) onto input d(TTGGGG)n primer oligonucleotide [5]. While TERC (located at 3q26.3) is widely expressed in cells and provides the template for d(TTGGGG) repeat synthesis, TERT (located at chromosome band 5p15.33) forms the catalytic subunit d(TTGGGG) and is far more regulated with limited/absent presents in somatic cells [5, 10]. The specific 5'-TTAGGG-3' sequence ending in a 3' single-stranded overhang ensures genomic DNA stability and protection from the continuous erosion process during the cell division cycle due to the G-strand overhang [4]. Under normal circumstances, progressive telomere loss forms the basis for cellular senescence and limited cellular life span as telomerase only activates in highly proliferative cells (stem/germ-line) as opposed to normal somatic cells [11]. In contrast, the majority of human malignancies (up to 90%) show heightened TERT expression and telomerase activity [12, 13]. This distinction in telomerase function between normal and malignant cells ensures a continuous reconstruction process of telomeres which provides the doorway toward unlimited proliferation and cellular immortalization and is thus an important stepping stone in oncogenesis [14, 15]. Literature reports that telomere maintenance occurs in all types of malignant cells [9, 16, 17] predominantly due to the upregulation of the telomerase enzyme (85–90%), which adds hexanucleotide repeats onto the ends of telomeric DNA. However, it can also be achieved using specific activation mechanisms based on interchromosomal exchanges of sequence information [18].

Over the years, research has shown how both telomere and telomerase bear influence over a wide number of human afflictions including cancer. Ever since Harley et al. [19] first proposed the theory that telomere shortening constitutes a determinant in the pathobiology of human disease. However, the TERT gene and its polymorphisms have yet to be thoroughly studied in terms of laryngeal cancer evolution and development. This is the first study to evaluate the relationship between the Rs2736100 (A>C) polymorphism of the TERT gene and laryngeal squamous cell carcinoma and correlate them with the clinical and pathological characteristics of this pathology.

## MATERIAL AND METHODS

### Subjects and biological samples

The study enrolled 56 LSCC subjects from the ENT Department of Cluj County Emergency Clinical Hospital, Romania, in 2018, from whom biological samples were collected to perform genetic testing. The samples consisted of 5 ml peripheral venous blood stored in Ethylenediaminetetraacetic acid (EDTA) coated tubes at -20℃ and a solid tumor fragment from archival formalin-fixed paraffin-embedded (FFPE) tissue samples. Recorded clinical data from each patient consisted of age, gender, and daily consumption rate of cigarettes. Tumors were classified according to the World Health Organization (WHO) Classification of Head and Neck Tumours and the NCCN (National Comprehensive Cancer Network) guidelines for head and neck cancers [2, 20]. The smoking index was calculated using the following formula: the number of cigarettes per day (CPD) multiplied by the number of smoking years [21, 22]. Subjects were then defined: non-smokers, mild (<400 cigarettes/day*year), moderate (400–800 cigarettes/day*year), severe smokers (>800 cigarettes/ day*year), and former smokers.

### DNA extraction and PCR analysis of TERT Rs2736100

Genetic testing was carried out on the samples of genetic material using Real-time-PCR genotyping assays [23] with commercial DNA extraction kits specifically designed for each type of sample: Wizard Genomic DNA Purification Kit and DNA extraction Kit PureLink Thermo Fisher Scientific Biogenetics Genetic All. The amount of DNA obtained by the two methods of extraction was then measured using an Eppendorf spectrophotometer so that only the samples with a DNA concentration of 10 micrograms/microl were subsequently used in the final genetic analysis. PCR amplification required the use of the following primer (GAAAAGCAGGGCGGGGGCACAAGCTA [A/C] AGAAACACTCAACACGGAAAACAAT) and specific thermocycling conditions consisting of initial DNA denaturation at 95℃ for 10 minutes followed by 40 reaction cycles for 15 seconds at 92℃ and for 1 minute at 60℃. Afterward, three variants were then identified using BioRad CFX96 Real-Time PCR Detection System software (Bio-Rad, Hercules, CA) and gel electrophoresis. They were: wild-type genotype (AA), heterozygote genotype (AC), and mutant homozygote genotype (CC).

### Statistical analysis

The resulting data was initially subjected to descriptive analysis according to the following criteria: gender, age groups (mean value), tumor classification criteria, histological grade, and smoking habits. Subjects were subsequently divided into the categories mentioned above according to the three genotypes identified for Rs2763100. The statistical examination was then performed to assess the possible influence each genotype had on the above-mentioned clinical and pathological variables using tests such as the Chi-square test, Chi-Square for Goodness of Fit, and Fisher's exact test. Analysis was carried out using Epi Info 7 and the Excel Data Analysis package with P-values<0.05 considered significant.

## RESULTS

### Clinical and pathological features of LSCC subjects

The clinical characteristics of the subjects, such as age, gender, and smoking habits, were recorded and assessed in univariate analysis (Table 1).

**Table 1:** Distribution of patients according to demographic characteristics.

Variables	Subjects
**Gender**	
Male	53
Female	3
**Age**	
Range	37–83
Mean age±SD	62.5±10.4 years
<62	25
≥62	31
**Smoking index**	
Non-smokers	4
<400 cigarettes/day*year	13
400–799 cigarettes/day*year	19
>800 cigarettes/day*year	18
Former smoker	2

Smoking index categories: mild (<400 cigarettes/day*year), moderate (400–800 cigarettes/day*year), severe smokers (>800 cigarettes/day*year).

Subjects were predominantly male (95% *vs*. 5%), with ages ranging from 37–83 years with a mean age of 62.5±10.4 years. Among the smoking population (89%), most subjects were in the higher risk groups, such as moderate and severe (38% and 36%, respectively), as opposed to mild smokers (26%), according to the smoking index categories. Distribution according to location, tumor classification criteria (pT, pN0, WHO grade), and degree of histological differentiation was recorded in Table 2.

**Table 2:** Distribution of subjects according to tumor classification criteria.

Primary tumor site	
Left Hemilarynx	14
Right Hemilarynx	11
Glottis	19
Supraglottis	8
Subglottis	1
Invasive towards the pharynx	3
**Histological grade**	
Well-differentiated	7
Moderately differentiated	36
Poorly differentiated	6
Other ^b^	7
**Primary tumor**	
T1	9
T2	4
T3	18
T4	25
**Lymph node metastasis**	
N0	39
N1	6
N2	11
N3	0
**WHO grade**	
I-II	10
III-Iv	46

T – the primary tumour; N – regional node metastasis; ^b^ – includes mixed histological grades such as invasive squamous cell carcinoma, unspecified histological grade.

The most common origin site was the glottis with 19 cases (34%), followed by left and right hemilarynx, respectively [14 (25%) and 11 (20%)]. Histological grade consisted of 36 (64%) moderately differentiated carcinoma (G2), 7 (13%) well-differentiated (G1), and 6 (11%) poorly differentiated (G3). The predominant clinical stage was III-IV with 46 (82%) as opposed to 10 (18%) I-II. Lymph node metastasis was absent in 39 (69%) patients and found in 17(31%).

### Rs2736100 genotype analysis

Among the three genetic variants of TERTRs2736100, the CC variant was the most common (20 cases, 36%), followed by AC (19, 34%) and AA (17, 30%). At the same time, no distinction between the genotypes from the DNA extracted from the paired genetic samples (100% matching samples) was found (Table 3).

**Table 3:** Distribution of TERT Rs2736100 genotypes in blood and tissue samples.

Nr.	Blood samples	Tissue samples
1	AA	AA
2	AA	AA
3	AC	AC
4	AC	AC
5	CC	CC
6	AC	AC
7	CC	CC
8	CC	CC
9	AC	AC
10	AA	AA
11	AA	AA
12	AC	AC
13	CC	CC
14	CC	CC
15	CC	CC
16	AA	AA
17	AC	AC
18	AC	AC
19	CC	CC
20	AC	AC
21	CC	CC
22	CC	CC
23	AC	AC
24	AA	AA
25	AA	AA
26	AC	AC
27	CC	CC
28	CC	CC
29	AA	AA
30	AA	AA
31	AA	AA
32	AC	AC
33	AC	AC
34	CC	CC
35	AC	AC
36	CC	CC
37	CC	CC
38	AC	AC
39	AA	AA
40	AA	AA
41	AC	AC
42	CC	CC
43	AA	AA
44	AC	AC
45	CC	CC
46	CC	CC
47	CC	CC
48	CC	CC
49	AC	AC
50	AA	AA
51	AC	AC
52	CC	CC
53	AC	AC
54	AA	AA
55	AA	AA
56	AA	AA

Rs2736100 polymorphism genotypes: wild-type variant (AA), mutant heterozygote AC, and mutant homozygote (CC).

Subsequent divisions of the three genotypes according to the predefined categories were recorded in Table 4.

**Table 4:** Distribution of TERT Rs2736100 genotypes according to the predefined categories.

Category	Variables	AA	AC	CC
**Age groups**	<62	6	9	10
	≥62	11	10	10
**Gender**	F	1	1	1
	M	16	18	19
**T**	T1	5	2	2
	T2	2	1	1
	T3	7	6	5
	T4	4	10	11
**N**	N0	16	12	11
	N1	0	2	4
	N2	1	5	5
**HG**	G1	4	1	2
	G2	8	15	13
	G3	2	1	3
**Smoking index**	Non-smokers	1	3	0
	Mild smokers	3	6	4
	Moderate smokers	5	7	7
	Severe smokers	6	3	9
	Former smoker	2	0	0

T – the primary tumour; N – regional node metastasis; HG – histological grade; G1 – well-differentiated squamos cell carcinoma; G2 – moderately differentiated squamos cell carcinoma; G3 – poorly differentiated squamos cell carcinoma; smoking index categories: mild (<400 cigarettes/day*year), moderate (400–800 cigarettes/day*year), severe smokers (>800 cigarettes/day*year).

Both CC and AC variants had the highest frequency in males, 36% and 34%, respectively, as opposed to AA (30%). Age-wise, all three genotypes favored subjects in the ≥62 years group, with the wild-type variant being the most common (36%). Distribution according to the tumor classification criteria highlights the association between the genetic variants and the different categories. All three genotypes were more common in the more advanced clinical stages (T3-T4) with moderately differentiated squamous cell carcinoma (G2) and a lack of regional lymph node metastasis (N0). Among smokers, the mutant variants of TERTRs2736100 were more frequent in subjects with a history of smoking between 20–39 years as opposed to the wild-type genotype, with little difference between them (42% AC, 41% CC, 17% AA). Secondary distribution according to the smoking index also identified the respective genotypes with higher frequencies: moderate and severe smokers for both AC and CC (7 cases each) and severe smokers for CC (9 cases).

### Statistical analyses

To correlate the genotypes of RS2736100 polymorphism with the recorded clinical and pathological features, we deferred to statistical tests such as Chi-square for goodness of fit, Chi-square test, and Fisher's exact test for statistical analysis. For this purpose, we defined the following comparison groups (Table 5) and divided the subjects according to each of the three genotypes: gender (male *vs*. female), age groups (<62 *vs*. ≥62), tumor classification criteria (T1-T2 *vs*. T3-T4; N0 *vs*. N1-N2), histological grade (well/moderate differentiated *vs*. poorly differentiated), supplementary histological risk criteria (SHC major *vs*. SHC minor), and smoking index.

**Table 5:** Statistical analysis of TERT Rs2736100 genotypes.

Category	P-value
**AA genotype**	
Gender	0.90825
Age groups	0.35282
T1-T2 vs. T3-T4	0.5586
N0 vs. N1-N2	**0.008541 ^c^**
G1-G2 vs. G3	0.78284
SHC major vs. SHC minor	**0.000016 ^c^**
**AC genotype**	
Gender	0.98214
Age groups	0.76875
T1-T2 vs. T3-T4	0.34652
N0 vs. N1-N2	0.44945
G1-G2 vs. G3	0.32203
SHC major vs. SHC minor	**0.000701 ^c^**
**CC genotype**	
Gender	0.929504
Age groups	0.54779
T1-T2 vs. T3-T4	0.34652
N0 vs. N1-N2	0.07568
G1-G2 vs. G3	0.471828
SHC major vs. SHC minor	**0.000117 ^c^**

SHC parameters: positive lymphatic invasion (L1), positive venous invasion (V1), positive resection margin (R1), perineural invasion (PN1), extranodal extension (ENE-). SHC major vs. minor categories: (R1 and ENE-) vs. (V1, L1, Pn1, N1-2). Bold values as statistically significant after application of FDR correction, p<0.05.

Although the CC variant showed the highest frequency among patients, no statistically significant domination was observed for the genotype in question as opposed to the other two variants when applying the Chi-square for goodness of fit test (P=0.982308). We then tested the individual variants of RS 2736100 against the above-mentioned categories but found no statistically significant results within any category for variants AC or CC (Table 5). Only one statistically significant result was noted in the association between the wild-type variant (AA) and the lack of lymph node metastasis N0 (P=0.0106). Assessment according to SHC (supplementary histological risk criteria) found that all three genotypes show a preference in the distribution of these parameters towards the low-risk factors such as negative lymphatic invasion (L0), negative venous invasion (V0) and negative resection margin (R0): AA, P=0.000866; AC, P=0.000016; CC, P=0.00001. Subsequent grouping of the genotypes according to the major and minor risk categories of SHC found all genotypes to have positive associations with the minor risk category (AA, P=0.000016; AC, P=0.000701; CC, P=0.000117). Among smokers, results could not confirm that both the number of years spent smoking and the number of cigarettes smoked influenced the genotype distribution (P=0.230177, P=0.498794, respectively). We then assessed the individual genotypes according to both categories in order to identify a possible dominant risk group and found positive associations for all three variants: AA/>40 years smoking P=0.00001, >800 cigarettes/day*year, P=0.00001; AC/20–39 years smoking P=0.00001, 400–799 cigarettes/day*year, P=0.00001; CC/20–39 years smoking P=0.000027, >800 cigarettes/day*year, P=0.000084)

## DISCUSSION

Many studies have been carried out over the years to identify the intimate mechanisms involved in malignant transformation and development. The ability to characterize individual human cancers at a genetic level has been a prime focus target in cancer research over the years. Literature reports positive associations between both cancerous and non-cancerous diseases concerning telomeres and the TERT gene due to either dysfunction or abnormal reactivations of telomerase activity. In a review, Calado et al. [24] reported how the various diseases of telomeres can induce severe pathologies such as idiopathic pulmonary fibrosis, bone marrow failure, and acquired aplastic anemia. Cod et al. [25] researched the TERT gene locus by studying genetic association databases for diseases and reported how the relationship between alleles and telomere length showed associations with idiopathic pulmonary fibrosis and specific cancer types. When referring to HNC, Hohaus et al. [26] performed a topographical analysis on laryngeal cancer that reported a pattern of telomerase activation within the tumor samples while correlating those results with the clinical stages. TERTRs2736100, however, continues to remain an unknown variable within this pathology. To date, studies report a positive risk association between the Rs2736100 polymorphism of the TERT gene and specific malignancies such as lung, colorectal, esophagus, and thyroid [5, 27, 28].

To our knowledge, this is the first study to investigate the role TERTRs2736100 may have concerning LSCC development. According to literature reports, the process of tumor development proceeds via a complex process with multiple steps in which successive genetic changes lead to the progressive conversion of normal cells into cancer cells [14, 15]. As mutations within malignant cells are a fairly common occurrence, we began this investigation by hypothesizing that the variants of Rs2736100 could suffer additional mutations within the cancer cells themselves as opposed to non-cancerous cells taken from the same individual. To test this hypothesis, we compared two different biological samples consisting of normal cells (blood sample) and cancer cells (squamous cell carcinoma tissue sample). The obtained results, as noted in Table 3, did not reveal any somatic mutation in the TERTRs2736100 variants. This result is consistent with the expected outcome of this analysis, as constitutional DNA is supposed to match the DNA found in somatic cells. We then proceeded to match the frequency of each of the three identified genotypes according to the specific predefined categories (Table 4) on which statistical examination was carried out. Results regarding the frequency of both gender and age distribution were per literature data, as laryngeal cancers tend to favor male subjects between 40 and 60 years of age, at least twice as often as females [2, 29]. Both primary tumor localization and histological differentiation were per known data, as most SCCs' of the larynx were proven to be moderately differentiated [2]. Studies regarding the site of origin for LSCC performed in the US report a predominance for the glottis (51%), followed by the supraglottis in 33% and the subglottis in 2% [30, 31]. In their work, Ferlito et al. [32] reported far better prognostic for glottic SCC as opposed to subglottic and hypopharyngeal SCC, which had the worst prognostic value.

Our results into the genotype distribution of TERTRs2736100 according to the tumor classification criteria could not confirm the association with a specific origin site in terms of a better prognosis. Although the majority of cases had a preference for the advanced clinical stage, only subjects carrying the normal wild-type genotype (AA) variant had a positive association with the lack of lymph node metastasis (N0). Despite this singular result, all three genotypes were also shown to favor better histological presentation regarding the pattern of tumor invasion, an outcome that tends to further promote the AA variant in terms of a better prognostic for LSCC compared to the other two genotypes.

Studies over the years have confirmed smoking as a leading risk factor in laryngeal cancer development in Western countries [33], with a minimum of 5–30 years of smoking required [8, 34]. Our results on smoking habits corroborate this notion. Although no individual risk was identified for the variants of this polymorphism, the added presence of cigarette smoking confirmed the characteristic increase in carcinogenic risk associated with that particular carcinogenic factor. All three genotypes exhibited synergistic relationships with subjects who were moderate to severe smokers according to both yearly consumption rates as well as the recorded smoking index categories, further promoting the notion that smoking remains one of the most crucial risk factors in LSCC development regardless of inherited genetic makeup.

## CONCLUSION

The results of our study suggest that the wild-type genotype AA of TERTRs2736100 may pose a protective factor in terms of lymph node metastasis as well as the histological pattern of tumor invasion in LSCC. Results regarding the synergistic relationship between cancer and smoking corroborate literature data on the subject for moderate to severe smokers regardless of the tested genetic variant.

## Personal thanks

We thank the Departments of Otolaryngology-Head and Neck Surgery, and Pathology of Cluj County Emergency Clinical Hospital and the Department of Genetics from the Iuliu Hatieganu University of Medicine and Pharmacy Cluj-Napoca, Romania, for their participation in realizing this study.
